# The genome sequence of the European Corn Borer, *Ostrinia nubilalis *Hübner, 1796

**DOI:** 10.12688/wellcomeopenres.23504.1

**Published:** 2025-01-14

**Authors:** Douglas Boyes, David C. Lees, Brad S. Coates, University of Oxford and Wytham Woods Genome Acquisition Lab, Natural History Museum Genome Acquisition Lab, Darwin Tree of Life Barcoding collective, Wellcome Sanger Institute Tree of Life Management, Samples and Laboratory team, Wellcome Sanger Institute Scientific Operations: Sequencing Operations, Wellcome Sanger Institute Tree of Life Core Informatics team, Tree of Life Core Informatics collective, Darwin Tree of Life Consortium

**Affiliations:** 1 UK Centre for Ecology & Hydrology, Wallingford, England, UK; 2 Natural History Museum, London, England, UK; 3 Corn Insects and Crop Genetics Research, USDA, Ames, Iowa, USA

**Keywords:** Ostrinia nubilalis, European Corn Borer, genome sequence, chromosomal, Lepidoptera

## Abstract

We present a genome assembly from an individual female specimen of *Ostrinia nubilalis* (European Corn Borer; Arthropoda; Insecta; Lepidoptera; Crambidae). The genome sequence has a total length of 495.50 megabases. Most of the assembly (99.87%) is scaffolded into 32 chromosomal pseudomolecules, including the Z and W sex chromosomes. The mitochondrial genome has also been assembled and is 15.24 kilobases in length. Gene annotation of this assembly on Ensembl identified 16,780 protein-coding genes.

## Species taxonomy

Eukaryota; Opisthokonta; Metazoa; Eumetazoa; Bilateria; Protostomia; Ecdysozoa; Panarthropoda; Arthropoda; Mandibulata; Pancrustacea; Hexapoda; Insecta; Dicondylia; Pterygota; Neoptera; Endopterygota; Amphiesmenoptera; Lepidoptera; Glossata; Neolepidoptera; Heteroneura; Ditrysia; Obtectomera; Pyraloidea; Crambidae; Pyraustinae; *Ostrinia*; *Ostrinia nubilalis* Hübner, 1796 (NCBI:txid29057)

## Background

The European corn borer (ECB), *Ostrinia nubilalis*, is a small snout-nosed moth species in the family Crambidae, with an endemic range across Europe, western Asia and Mediterranean region of Africa. Introduction into North America during the early 1900s led to its eventual establishment across areas east of the Rocky Mountains of the United States, and maritime and southern tiers of central plain provinces in Canada. Larval *O. nubilalis* are reported to feed on over 240 host plants, including cultivated maize, a host plant that suffers significant yield losses ( Hodgson, 1928). Economic costs to farmers in North America were severe, exceeding 1 billion USD in control measures and yield losses ( Mason, 1996). After the commercialisation of transgenic maize hybrids with insecticidal *Bacillus thuringiensis* ( *Bt*) insecticidal crystalline (Cry) proteins in 1995, area-wide effects caused widespread population suppression and vastly reduced incidence of associated crop damage ( Hutchison *et al.*, 2010). After nearly 25 years of successful suppression, damage was reported in Canada on transgenic maize expressing *Bt* Cry1F ( Smith *et al.*, 2019) and Cry1Ab proteins ( Smith & Farhan, 2023).

Like its phylogenetic sister and sibling species, *O. furnacalis* and *O. scapulalis*, respectively, male and female *O. nubilalis* are sexually dimorphic, with males having smaller body size and darker colouration compared to females ( Mutuura & Munroe, 1970). As in other species of Lepidoptera, *Ostrinia* females are heterogametic (ZW) and males are homogametic (ZZ). Ecological variation exists within and among *O. nubilalis* populations based on differences in sex pheromone produced by females and responded to by cognate males, and response to environmental cues that determine the number of reproductive generations per year ( Coates *et al.*, 2018). Specifically, E-(NY strain) and Z-race (IA strain) females produce stereoisomers *E*- and *Z*-tetradecyl acetate ( *E*-11Ac and *Z*-11Ac), respectively ( Klun & Maini, 1979), which is controlled by allelic variation in the autosomal gene, pheromone gland fatty acyl-reductase ( *pgfar*; Lassance *et al*., 2013). E- and Z-races are sympatric across the northern Atlantic coast of North America, whereas the geographic range of Z-race also extends westward across major maize growing regions of the United States ( Lambden & Johnson, 2013). Corresponding preferential male attraction is determined by variation in neuronal development that is under direction of E- and Z-race specific differences in the sex-linked transcription factor, *bric à brac* ( *bab*) ( Unbehend *et al.*, 2021). The number of mating generations (voltinism) varies across latitudinal clines, with genetic determination of obligatory diapause among fixed single generation univoltine populations in the northern regions. Phenotypic plasticity in a facultative diapause trait exists among multivoltine individuals, resulting in up to five mating generations in the southern regions ( Showers, 1993). These voltine differences are controlled by epistatic interactions between allelic variants of the sex-linked genes period ( *per*) and pigment dispersing factor receptor ( *pdfr*), that are part of circadian and circannual rhythm pathways, respectively ( Kozak *et al.*, 2019). Additionally, an approximate 50 Mbp region of suppressed recombination, likely an inversion, occurs on the Z-chromosome of *O. nubilalis* that includes *per*, with *bab* and *pdfr* located in Z-chromosome regions flanking predicted breakpoints ( Coates *et al.*, 2018; Kozak *et al.*, 2017). Interestingly, this likely inverted region has an elevated frequency within sympatric populations that differ in one or more voltine and/or pheromone traits, suggesting a role in reinforcing assortative mating and persistence of ecotypes.

A chromosome-level genome assembly was generated for *O. nubilalis* as part of the Darwin Tree of Life project. This assembly will contribute toward the growing resources for studying evolution across Lepidoptera, as well as ecological adaptations and underlying genomic differentiation in the genus *Ostrinia*.

## Genome sequence report

**Figure 1.**
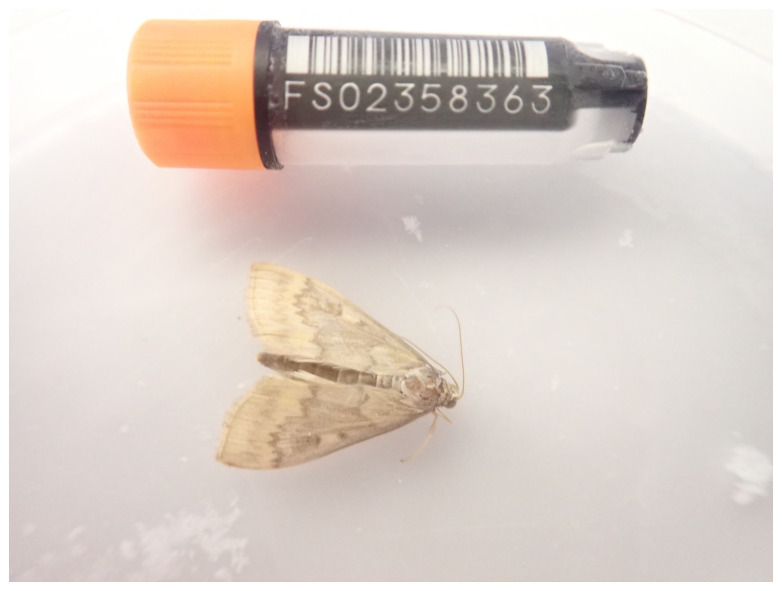
Photograph of the *Ostrinia nubilalis* (ilOstNubi1) specimen used for genome sequencing.

The genome of *Ostrinia nubilalis* ( Figure 1) was sequenced using Pacific Biosciences single-molecule HiFi long reads, generating a total of 21.96 Gb (gigabases) from 1.50 million reads, providing an estimated 44-fold coverage. Primary assembly contigs were scaffolded with chromosome conformation Hi-C data, which produced 106.66 Gb from 706.37 million reads. Specimen and sequencing details are summarised in Table 1.

**Table 1.** Specimen and sequencing data for *Ostrinia nubilalis*.

Project information			
**Study title**	Ostrinia nubilalis (European corn borer)		
**Umbrella BioProject**	PRJEB65201		
**Species**	*Ostrinia nubilalis*		
**BioSample**	SAMEA7701321		
**NCBI taxonomy ID**	29057		
Specimen information			
**Technology**	**ToLID**	**BioSample ** **accession**	**Organism part**
**PacBio long read ** **sequencing**	ilOstNubi1	SAMEA7701417	Whole organism
**Hi-C sequencing**	ilOstNubi4	SAMEA112975556	Whole organism
Sequencing information			
**Platform**	**Run accession**	**Read count**	**Base count (Gb)**
**Hi-C Illumina NovaSeq ** **6000**	ERR11872563	7.06e+08	106.66
**PacBio Sequel IIe**	ERR11867203	1.50e+06	21.96

Assembly errors were corrected by manual curation, including 12 missing joins or mis-joins and four haplotypic duplications. This increased the scaffold number by 1.92%. The final assembly has a total length of 495.50 Mb in 52 sequence scaffolds, with 17 gaps, and a scaffold N50 of 16.5 Mb ( Table 2).

**Table 2.** Genome assembly data for *Ostrinia nubilalis*, ilOstNubi1.1.

Genome assembly		
Assembly name	ilOstNubi1.1	
Assembly accession	GCA_963855985.1	
*Accession of alternate haplotype*	*GCA_963856005.1*	
Span (Mb)	495.50	
Number of contigs	70	
Number of scaffolds	52	
Longest scaffold (Mb)	27.76	
Assembly metrics *		*Benchmark*
Contig N50 length (Mb)	15.5	*≥ 1 Mb*
Scaffold N50 length (Mb)	16.5	*= chromosome N50*
Consensus quality (QV)	68.8 (68.4 for combined haplotypes)	*≥ 40*
*k*-mer completeness	Primary haplotype: 66.52%, alternate haplotype 61.67%, both 98.91%	*≥ 95%*
BUSCO v5.4.3 lineage: lepidoptera_odb10	C:98.5%[S:98.2%,D:0.3%], F:0.5%,M:1.0%,n:5,286	*S > 90%*, *D < 5%*
Percentage of assembly mapped to chromosomes	99.87%	*≥ 90%*
Sex chromosomes	ZW	*localised homologous * *pairs*
Organelles	Mitochondrial genome: 15.24 kb	*complete single alleles*
Genome annotation of assembly GCA_963855985.1 at Ensembl		
Number of protein-coding genes	16,780	
Number of non-coding genes	10,413	
Number of gene transcripts	45,650	

* Assembly metric benchmarks are adapted from Rhie *et al.* (2021) and the Earth BioGenome Project Report on Assembly Standards September 2024. ** BUSCO scores based on the lepidoptera_odb10 BUSCO set using version 5.4.3. C = complete [S = single copy, D = duplicated], F = fragmented, M = missing, n = number of orthologues in comparison. A full set of BUSCO scores is available at https://blobtoolkit.genomehubs.org/view/Ostrinia_nubilalis/dataset/GCA_963855985.1/busco.

**Figure 2.**
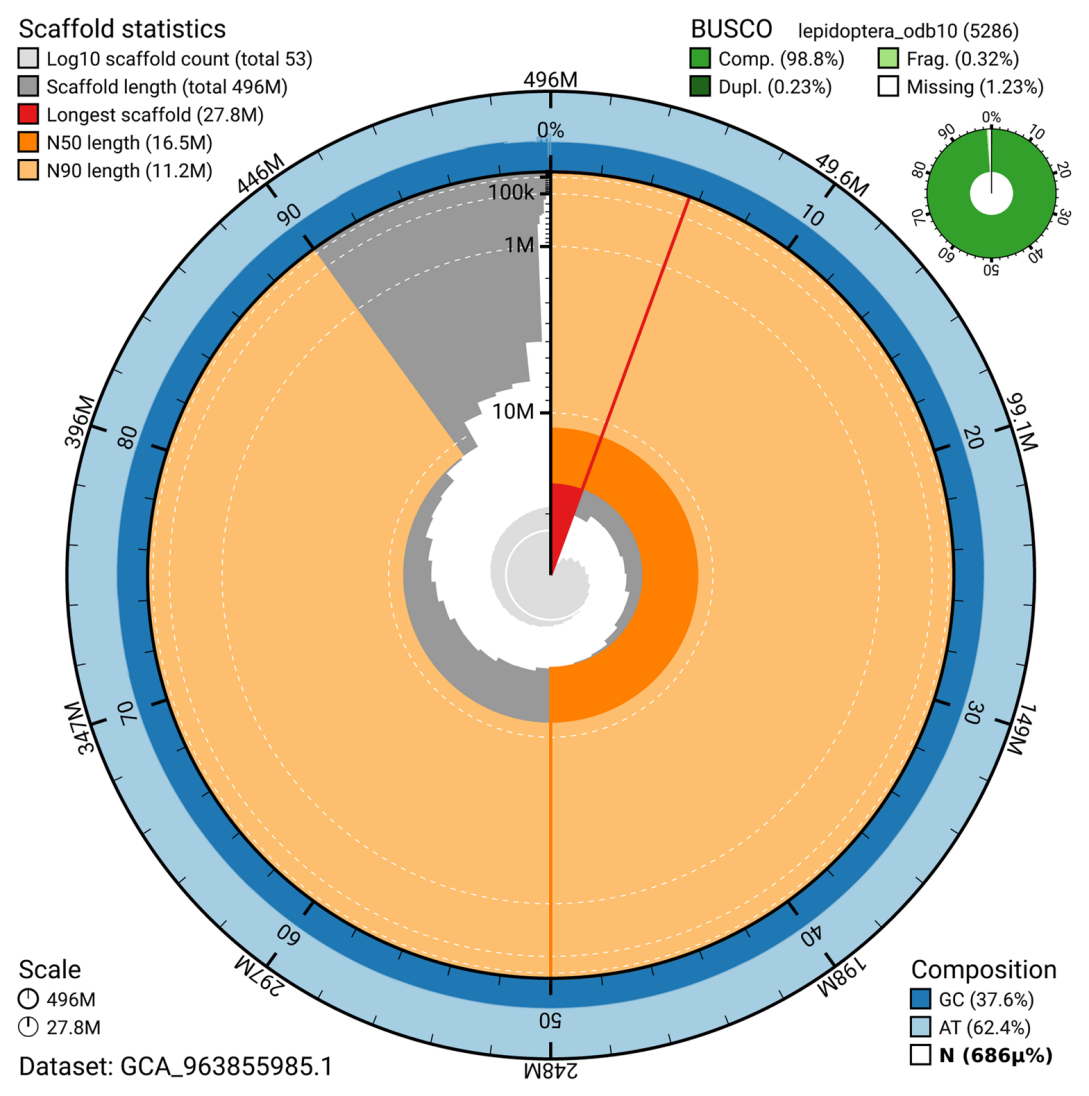
Genome assembly of *Ostrinia nubilalis*, ilOstNubi1.1: metrics. The BlobToolKit snail plot provides an overview of assembly metrics and BUSCO gene completeness. The circumference represents the length of the whole genome sequence, and the main plot is divided into 1,000 bins around the circumference. The outermost blue tracks display the distribution of GC, AT, and N percentages across the bins. Scaffolds are arranged clockwise from longest to shortest and are depicted in dark grey. The longest scaffold is indicated by the red arc, and the deeper orange and pale orange arcs represent the N50 and N90 lengths. A light grey spiral at the centre shows the cumulative scaffold count on a logarithmic scale. A summary of complete, fragmented, duplicated, and missing BUSCO genes in the lepidoptera_odb10 set is presented at the top right. An interactive version of this figure is available at https://blobtoolkit.genomehubs.org/view/GCA_963855985.1/dataset/GCA_963855985.1/snail.

**Figure 3.**
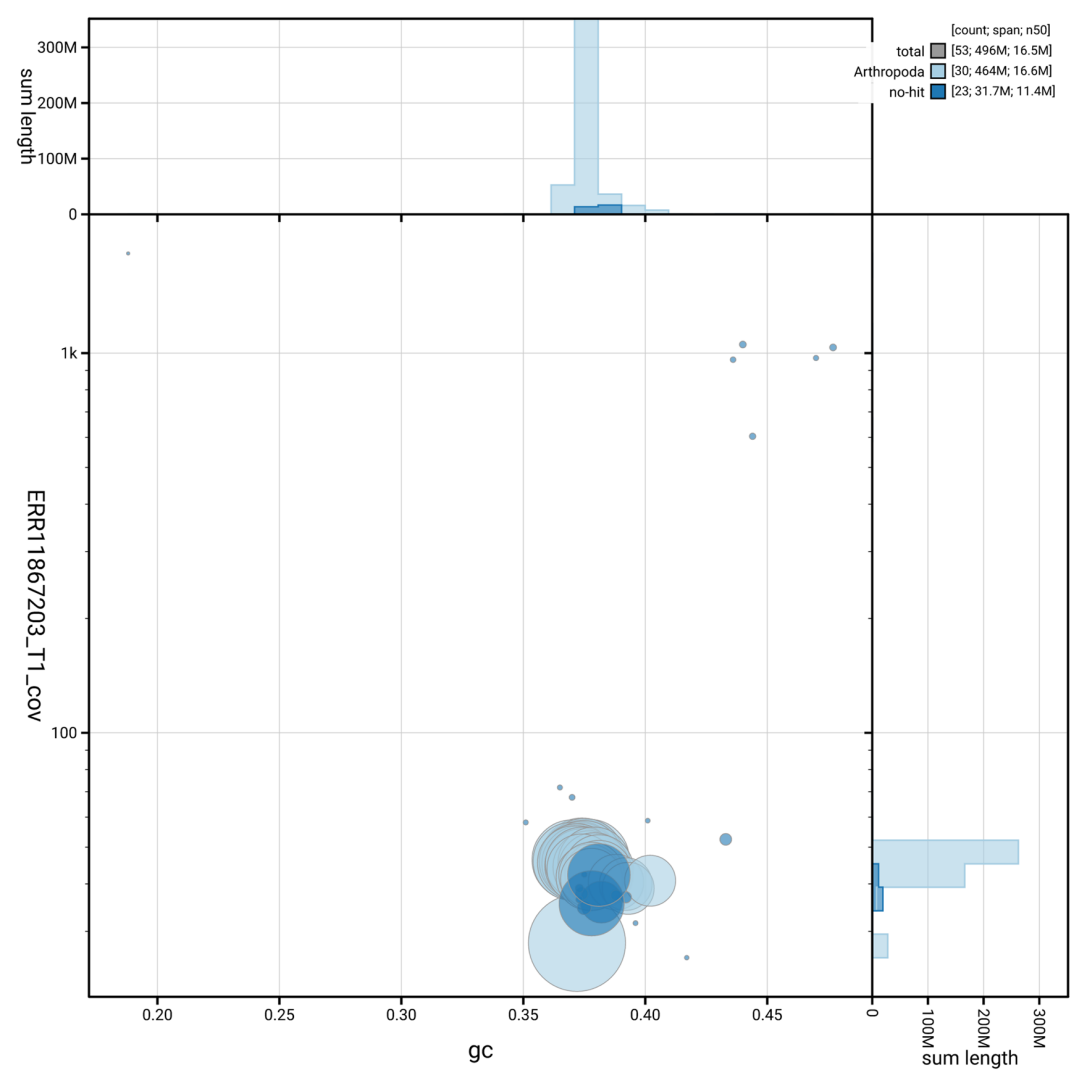
Genome assembly of *Ostrinia nubilalis*, ilOstNubi1.1: BlobToolKit GC-coverage plot showing sequence coverage (vertical axis) and GC content (horizontal axis). The circles represent scaffolds, with the size proportional to scaffold length and the colour representing phylum membership. The histograms along the axes display the total length of sequences distributed across different levels of coverage and GC content. An interactive version of this figure is available at https://blobtoolkit.genomehubs.org/view/GCA_963855985.1/dataset/GCA_963855985.1/blob.

**Figure 4.**
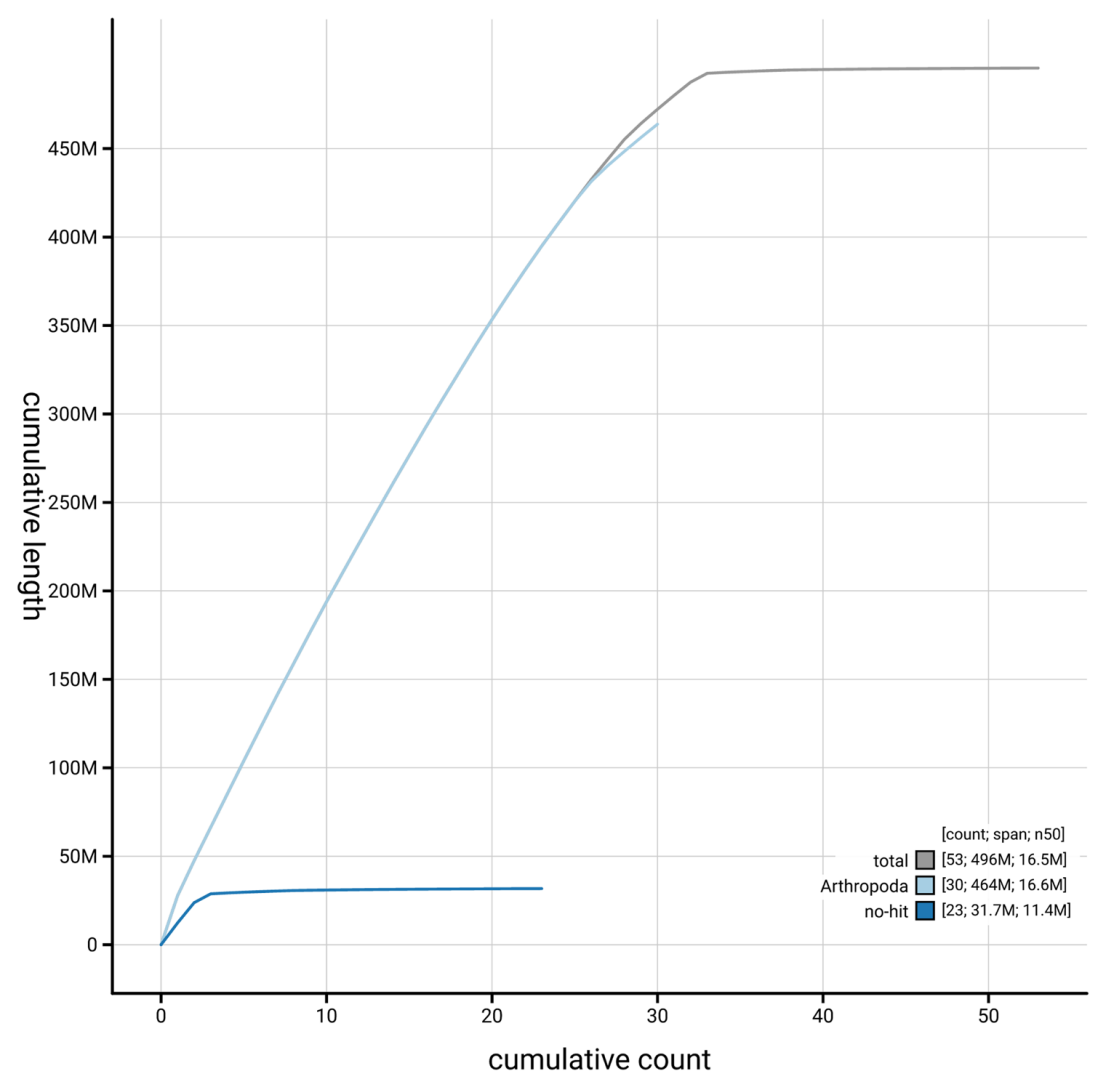
Genome assembly of *Ostrinia nubilalis* ilOstNubi1.1: BlobToolKit cumulative sequence plot. The grey line shows cumulative length for all scaffolds. Coloured lines show cumulative lengths of scaffolds assigned to each phylum using the buscogenes taxrule. An interactive version of this figure is available at https://blobtoolkit.genomehubs.org/view/GCA_963855985.1/dataset/GCA_963855985.1/cumulative.

The snail plot in Figure 2 provides a summary of the assembly statistics, indicating the distribution of scaffold lengths and other assembly metrics. Figure 3 shows the distribution of scaffolds by GC proportion and coverage. Figure 4 presents a cumulative assembly plot, with separate curves representing different scaffold subsets assigned to various phyla, illustrating the completeness of the assembly.

**Figure 5.**
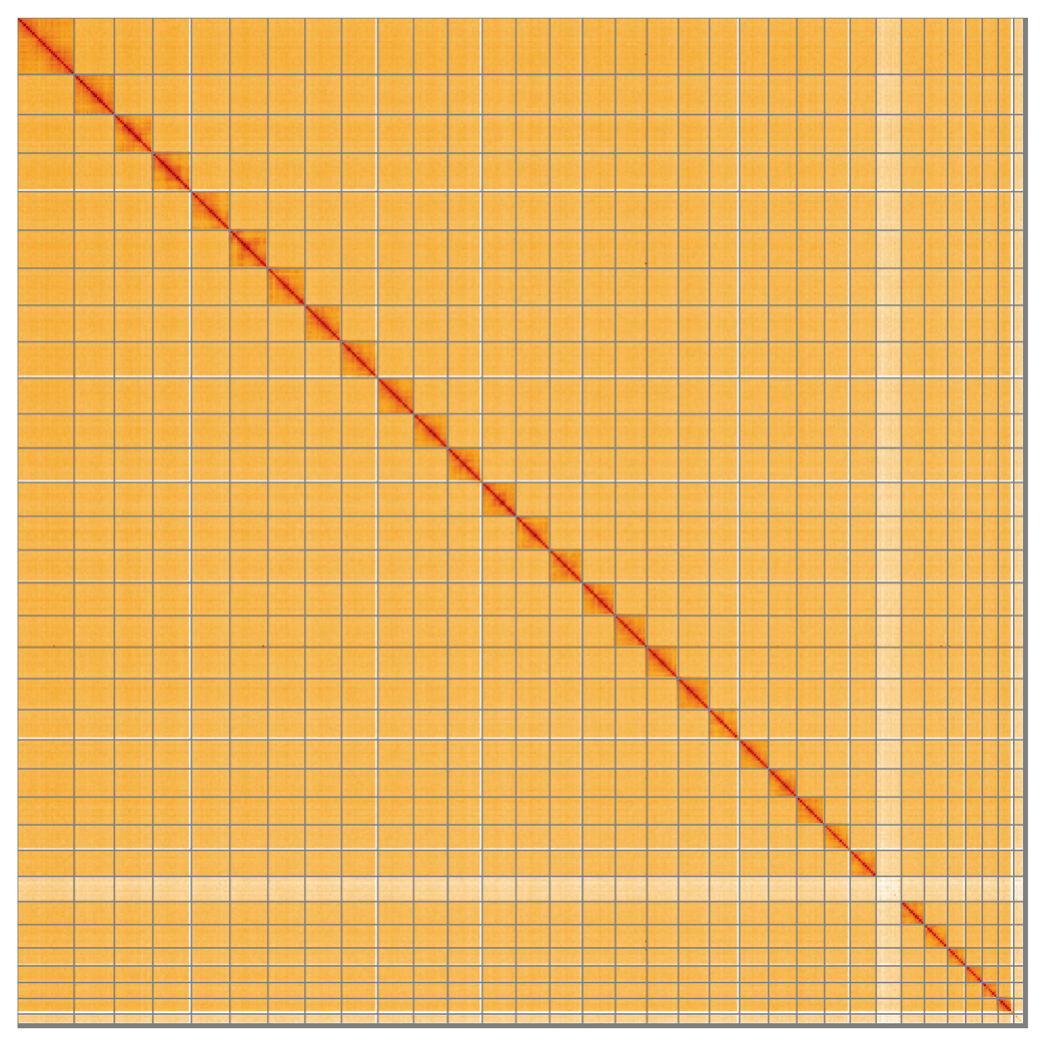
Genome assembly of *Ostrinia nubilalis* ilOstNubi1.1: Hi-C contact map of the ilOstNubi1.1 assembly, visualised using HiGlass. Chromosomes are shown in order of size from left to right and top to bottom. An interactive version of this figure may be viewed at https://genome-note-higlass.tol.sanger.ac.uk/l/?d=DXu8jeAmRwO2saQMFKY0Gg.

Most of the assembly sequence (99.87%) was assigned to 32 chromosomal-level scaffolds, representing 30 autosomes and the Z and W sex chromosomes. These chromosome-level scaffolds, confirmed by the Hi-C data, are named in order of size ( Figure 5; Table 3). During manual curation it was noted that The Hi-C data was from a male sample, so the exact order of the W chromosome contigs is unknown. The sex chromosomes were identified by coverage.

**Table 3.** Chromosomal pseudomolecules in the genome assembly of *Ostrinia nubilalis*, ilOstNubi1.

INSDC accession	Name	Length (Mb)	GC%
OY979734.1	1	19.71	37.5
OY979736.1	2	18.86	37.5
OY979737.1	3	18.82	37.5
OY979738.1	4	18.81	37.0
OY979739.1	5	18.55	37.5
OY979740.1	6	18.36	37.5
OY979741.1	7	17.84	37.5
OY979742.1	8	17.8	37.0
OY979743.1	9	17.37	37.5
OY979744.1	10	16.89	37.5
OY979745.1	11	16.74	37.0
OY979746.1	12	16.57	37.0
OY979747.1	13	16.46	37.5
OY979748.1	14	16.09	37.0
OY979749.1	15	16.03	37.5
OY979750.1	16	15.55	37.5
OY979751.1	17	15.27	37.5
OY979752.1	18	15.24	38.0
OY979753.1	19	14.73	38.0
OY979754.1	20	14.23	37.5
OY979755.1	21	13.82	38.0
OY979756.1	22	13.44	38.0
OY979757.1	23	12.75	37.5
OY979758.1	24	12.57	38.0
OY979759.1	25	11.41	38.0
OY979760.1	26	11.24	38.0
OY979761.1	27	8.94	39.0
OY979762.1	28	8.05	39.0
OY979763.1	29	7.82	39.5
OY979764.1	30	7.5	40.0
OY979735.1	W	12.36	38.0
OY979733.1	Z	27.76	37.0
OY979765.1	MT	0.02	19.0

While not fully phased, the assembly deposited is of one haplotype. Contigs corresponding to the second haplotype have also been deposited. The mitochondrial genome was also assembled and can be found as a contig within the multifasta file of the genome submission, and as a separate fasta file with accession OY979765.1.

The final assembly has a Quality Value (QV) of 68.8. The *k*-mer completeness of the primary assembly was found to be 66.52%, and of the alternate haplotype 61.67%, while the *k*-mer completeness for combined assemblies was 98.91%. BUSCO (v5.4.3) analysis using the lepidoptera_odb10 reference set ( *n* = 5,286) indicated a completeness score of 98.5% (single = 98.2%, duplicated = 0.3%). The assembly achieves the EBP reference standard of 6.7.68. Other quality metrics are given in Table 2.

## Genome annotation report

The *Ostrinia nubilalis* genome assembly (GCA_963855985.1) was annotated at the European Bioinformatics Institute (EBI) on Ensembl Rapid Release. The resulting annotation includes 45,650 transcribed mRNAs from 16,780 protein-coding and 10,413 non-coding genes ( Table 2; https://rapid.ensembl.org/Ostrinia_nubilalis_GCA_963855985.1/Info/Index). The average transcript length is 13,875.99. There are 1.68 coding transcripts per gene and 5.80 exons per transcript.

## Methods

### Sample acquisition and DNA barcoding

An adult female specimen of *Ostrinia nubilalis* (specimen ID Ox000555, ToLID ilOstNubi1) was collected from Wytham Woods, Berkshire, United Kingdom (latitude 51.77, longitude –1.32) on 2020-06-25, using a light trap. The specimen was collected and identified by Douglas Boyes (University of Oxford) and preserved on dry ice.

The specimen used for Hi-C sequencing (specimen ID NHMUK014584837, ToLID ilOstNubi4) was an adult specimen collected from Lucas Road, High Wycombe, England, United Kingdom (latitude 51.63, longitude –0.74) on 2022-06-23. The specimen was collected and identified by David Lees (Natural History Museum) and preserved by dry freezing at –80 °C.

The initial identification was verified by an additional DNA barcoding process according to the framework developed by Twyford *et al*. (2024). A small sample was dissected from the specimens and stored in ethanol, while the remaining parts were shipped on dry ice to the Wellcome Sanger Institute (WSI). The tissue was lysed, the COI marker region was amplified by PCR, and amplicons were sequenced and compared to the BOLD database, confirming the species identification ( Crowley *et al.*, 2023). Following whole genome sequence generation, the relevant DNA barcode region was also used alongside the initial barcoding data for sample tracking at the WSI ( Twyford *et al.*, 2024). The standard operating procedures for Darwin Tree of Life barcoding have been deposited on protocols.io ( Beasley *et al.*, 2023).

### Nucleic acid extraction

The workflow for high molecular weight (HMW) DNA extraction at the Wellcome Sanger Institute (WSI) Tree of Life Core Laboratory includes a sequence of procedures: sample preparation and homogenisation, DNA extraction, fragmentation and purification. Detailed protocols are available on protocols.io ( Denton *et al.*, 2023b). The ilOstNubi1 sample was prepared for DNA extraction by weighing and dissecting it on dry ice ( Jay *et al.*, 2023). Tissue from the whole organism was homogenised using a PowerMasher II tissue disruptor ( Denton *et al.*, 2023a).

HMW DNA was extracted using the Automated MagAttract v1 protocol ( Sheerin *et al.*, 2023). DNA was sheared into an average fragment size of 12–20 kb in a Megaruptor 3 system ( Todorovic *et al.*, 2023). Sheared DNA was purified by solid-phase reversible immobilisation, using AMPure PB beads to eliminate shorter fragments and concentrate the DNA ( Strickland *et al.*, 2023). The concentration of the sheared and purified DNA was assessed using a Nanodrop spectrophotometer and a Qubit Fluorometer using the Qubit dsDNA High Sensitivity Assay kit. The fragment size distribution was evaluated by running the sample on the FemtoPulse system.

### Hi-C preparation

Tissue from the ilOstNubi4 sample was processed at the WSI Scientific Operations core, using the Arima-HiC v2 kit. Tissue (stored at –80 °C) was fixed, and the DNA crosslinked using a TC buffer with 22% formaldehyde. After crosslinking, the tissue was homogenised using the Diagnocine Power Masher-II and BioMasher-II tubes and pestles. Following the kit manufacturer's instructions, crosslinked DNA was digested using a restriction enzyme master mix. The 5’-overhangs were then filled in and labelled with biotinylated nucleotides and proximally ligated. An overnight incubation was carried out for enzymes to digest remaining proteins and for crosslinks to reverse. A clean up was performed with SPRIselect beads prior to library preparation.

### Library preparation and sequencing

Library preparation and sequencing were performed at the WSI Scientific Operations core. Pacific Biosciences HiFi circular consensus DNA sequencing libraries were prepared using the PacBio Express Template Preparation Kit v2.0 (Pacific Biosciences, California, USA) as per the manufacturer's instructions. The kit includes the reagents required for removal of single-strand overhangs, DNA damage repair, end repair/A-tailing, adapter ligation, and nuclease treatment. Library preparation also included a library purification step using AMPure PB beads (Pacific Biosciences, California, USA) and size selection step to remove templates shorter than 3 kb using AMPure PB modified SPRI. DNA concentration was quantified using the Qubit Fluorometer v2.0 and Qubit HS Assay Kit and the final library fragment size analysis was carried out using the Agilent Femto Pulse Automated Pulsed Field CE Instrument and 165kb gDNA and 55kb BAC analysis kit. Samples were sequenced using the Sequel IIe system (Pacific Biosciences, California, USA). The concentration of the library loaded onto the Sequel IIe was in the range 40–135 pM. The SMRT link software, a PacBio web-based end-to-end workflow manager, was used to set-up and monitor the run, as well as perform primary and secondary analysis of the data upon completion.

For Hi-C library preparation, DNA was fragmented to a size of 400 to 600 bp using a Covaris E220 sonicator. The DNA was then enriched, barcoded, and amplified using the NEBNext Ultra II DNA Library Prep Kit following manufacturers’ instructions. The Hi-C sequencing was performed using paired-end sequencing with a read length of 150 bp on an Illumina NovaSeq 6000 instrument.

### Genome assembly, curation and evaluation

***Assembly***

The HiFi reads were first assembled using Hifiasm ( Cheng *et al.*, 2021) with the --primary option. Haplotypic duplications were identified and removed using purge_dups ( Guan *et al.*, 2020). The Hi-C reads were mapped to the primary contigs using bwa-mem2 ( Vasimuddin *et al.*, 2019). The contigs were further scaffolded using the provided Hi-C data ( Rao *et al.*, 2014) in YaHS ( Zhou *et al.*, 2023) using the --break option for handling potential misassemblies. The scaffolded assemblies were evaluated using Gfastats ( Formenti *et al.*, 2022), BUSCO ( Manni *et al.*, 2021) and MERQURY.FK ( Rhie *et al.*, 2020), using a *k*-mer database ( *k* = 31) constructed using FastK.

The mitochondrial genome was assembled using MitoHiFi ( Uliano-Silva *et al.*, 2023), which runs MitoFinder ( Allio *et al.*, 2020) and uses these annotations to select the final mitochondrial contig and to ensure the general quality of the sequence.

***Assembly curation***

The assembly was decontaminated using the Assembly Screen for Cobionts and Contaminants (ASCC) pipeline (article in preparation). Flat files and maps used in curation were generated in TreeVal ( Pointon *et al.*, 2023). Manual curation was primarily conducted using PretextView ( Harry, 2022), with additional insights provided by JBrowse2 ( Diesh *et al.*, 2023) and HiGlass ( Kerpedjiev *et al.*, 2018). Scaffolds were visually inspected and corrected as described by Howe *et al*. (2021). Any identified contamination, missed joins, and mis-joins were corrected, and duplicate sequences were tagged and removed. The curation process is documented at https://gitlab.com/wtsi-grit/rapid-curation (article in preparation).

***Assembly quality assessment***

The MerquryFK tool ( Rhie *et al.*, 2020), run within a Singularity container ( Kurtzer *et al.*, 2017), was used to evaluate *k*-mer completeness and assembly quality for the curated haplotypes (Hap1/2), using the *k*-mer databases ( *k* = 31) that were pre-computed prior to genome assembly. The analysis outputs included assembly QV scores and completeness statistics.

Three Nextflow ( Di Tommaso *et al.*, 2017) DSL2 pipelines: sanger-tol/readmapping ( Surana *et al.*, 2023a), sanger-tol/genomenote ( Surana *et al.*, 2023b), and sanger-tol/blobtoolkit ( Muffato *et al.*, 2024). The readmapping pipeline aligns the Hi-C reads using bwa-mem2 ( Vasimuddin *et al.*, 2019) and combines the alignment files with SAMtools ( Danecek *et al.*, 2021). The genomenote pipeline converts the Hi-C alignments into a contact map using BEDTools ( Quinlan & Hall, 2010) and the Cooler tool suite ( Abdennur & Mirny, 2020). The contact map is visualised in HiGlass ( Kerpedjiev *et al.*, 2018).

The blobtoolkit pipeline is a Nextflow port of the previous Snakemake Blobtoolkit pipeline ( Challis *et al.*, 2020). It aligns the PacBio reads in SAMtools and minimap2 ( Li, 2018) and generates coverage tracks for regions of fixed size. In parallel, it queries the GoaT database ( Challis *et al.*, 2023) to identify all matching BUSCO lineages to run BUSCO ( Manni *et al.*, 2021). For the three domain-level BUSCO lineages, the pipeline aligns the BUSCO genes to the UniProt Reference Proteomes database ( Bateman *et al.*, 2023) with DIAMOND blastp ( Buchfink *et al.*, 2021). The genome is also divided into chunks according to the density of the BUSCO genes from the closest taxonomic lineage, and each chunk is aligned to the UniProt Reference Proteomes database using DIAMOND blastx. Genome sequences without a hit are chunked using seqtk and aligned to the NT database with blastn ( Altschul *et al.*, 1990). The blobtools suite combines all these outputs into a blobdir for visualisation.

The genome evaluation pipelines were developed using nf-core tooling ( Ewels *et al.*, 2020) and MultiQC ( Ewels *et al.*, 2016), relying on the Conda package manager, the Bioconda initiative ( Grüning *et al.*, 2018), the Biocontainers infrastructure ( da Veiga Leprevost *et al.*, 2017), as well as the Docker ( Merkel, 2014) and Singularity ( Kurtzer *et al.*, 2017) containerisation solutions.

Table 4 contains a list of relevant software tool versions and sources.

**Table 4.** Software tools: versions and sources.

Software tool	Version	Source
BEDTools	2.30.0	https://github.com/arq5x/bedtools2
BLAST	2.14.0	http://ftp.ncbi.nlm.nih.gov/blast/executables/blast+/
BlobToolKit	4.3.7	https://github.com/blobtoolkit/blobtoolkit
BUSCO	5.4.3 and 5.5.0	https://gitlab.com/ezlab/busco
bwa-mem2	2.2.1	https://github.com/bwa-mem2/bwa-mem2
Cooler	0.8.11	https://github.com/open2c/cooler
DIAMOND	2.1.8	https://github.com/bbuchfink/diamond
fasta_windows	0.2.4	https://github.com/tolkit/fasta_windows
FastK	427104ea91c78c3b8b8b49f1a7d6bbeaa869ba1c	https://github.com/thegenemyers/FASTK
Gfastats	1.3.6	https://github.com/vgl-hub/gfastats
GoaT CLI	0.2.5	https://github.com/genomehubs/goat-cli
Hifiasm	0.19.8-r587	https://github.com/chhylp123/hifiasm
HiGlass	44086069ee7d4d3f6f3f0012569789ec138f42b84 aa44357826c0b6753eb28de	https://github.com/higlass/higlass
Merqury.FK	d00d98157618f4e8d1a9190026b19b471055b22e	https://github.com/thegenemyers/MERQURY.FK
MitoHiFi	3	https://github.com/marcelauliano/MitoHiFi
MultiQC	1.14, 1.17, and 1.18	https://github.com/MultiQC/MultiQC
NCBI Datasets	15.12.0	https://github.com/ncbi/datasets
Nextflow	23.04.0-5857	https://github.com/nextflow-io/nextflow
PretextView	0.2.5	https://github.com/sanger-tol/PretextView
purge_dups	1.2.5	https://github.com/dfguan/purge_dups
samtools	1.16.1, 1.17, and 1.18	https://github.com/samtools/samtools
sanger-tol/ ascc	-	https://github.com/sanger-tol/ascc
sanger-tol/ blobtoolkit	0.6.0	https://github.com/sanger-tol/blobtoolkit
sanger-tol/ genomenote	1.1.1	https://github.com/sanger-tol/genomenote
sanger-tol/ readmapping	1.2.1	https://github.com/sanger-tol/readmapping
Seqtk	1.3	https://github.com/lh3/seqtk
Singularity	3.9.0	https://github.com/sylabs/singularity
TreeVal	1.0.0	https://github.com/sanger-tol/treeval
YaHS	1.2a.2	https://github.com/c-zhou/yahs

### Genome annotation

The Ensembl Genebuild annotation system ( Aken *et al.*, 2016) was used to generate annotation for the *Ostrinia nubilalis* assembly (GCA_963855985.1) in Ensembl Rapid Release at the EBI. Annotation was created primarily through alignment of transcriptomic data to the genome, with gap filling via protein-to-genome alignments of a select set of proteins from UniProt ( UniProt Consortium, 2019).

### Wellcome Sanger Institute – Legal and Governance

The materials that have contributed to this genome note have been supplied by a Darwin Tree of Life Partner. The submission of materials by a Darwin Tree of Life Partner is subject to the **‘Darwin Tree of Life Project Sampling Code of Practice’**, which can be found in full on the Darwin Tree of Life website here. By agreeing with and signing up to the Sampling Code of Practice, the Darwin Tree of Life Partner agrees they will meet the legal and ethical requirements and standards set out within this document in respect of all samples acquired for, and supplied to, the Darwin Tree of Life Project.

Further, the Wellcome Sanger Institute employs a process whereby due diligence is carried out proportionate to the nature of the materials themselves, and the circumstances under which they have been/are to be collected and provided for use. The purpose of this is to address and mitigate any potential legal and/or ethical implications of receipt and use of the materials as part of the research project, and to ensure that in doing so we align with best practice wherever possible. The overarching areas of consideration are:

•   Ethical review of provenance and sourcing of the material

•   Legality of collection, transfer and use (national and international)

Each transfer of samples is further undertaken according to a Research Collaboration Agreement or Material Transfer Agreement entered into by the Darwin Tree of Life Partner, Genome Research Limited (operating as the Wellcome Sanger Institute), and in some circumstances other Darwin Tree of Life collaborators.

## Data Availability

European Nucleotide Archive: Ostrinia nubilalis (European corn borer). Accession number PRJEB65201; https://identifiers.org/ena.embl/PRJEB65201. The genome sequence is released openly for reuse. The *Ostrinia nubilalis* genome sequencing initiative is part of the Darwin Tree of Life (DToL) project. All raw sequence data and the assembly have been deposited in INSDC databases. Raw data and assembly accession identifiers are reported in Table 1 and Table 2.

## Author information

Members of the University of Oxford and Wytham Woods Genome Acquisition Lab are listed here: https://doi.org/10.5281/zenodo.12157525.

Members of the Natural History Museum Genome Acquisition Lab are listed here: https://doi.org/10.5281/zenodo.12159242.

Members of the Darwin Tree of Life Barcoding collective are listed here: https://doi.org/10.5281/zenodo.12158331.

Members of the Wellcome Sanger Institute Tree of Life Management, Samples and Laboratory team are listed here: https://doi.org/10.5281/zenodo.12162482.

Members of Wellcome Sanger Institute Scientific Operations: Sequencing Operations are listed here: https://doi.org/10.5281/zenodo.12165051.

Members of the Wellcome Sanger Institute Tree of Life Core Informatics team are listed here: https://doi.org/10.5281/zenodo.12160324.

Members of the Tree of Life Core Informatics collective are listed here: https://doi.org/10.5281/zenodo.12205391.

Members of the Darwin Tree of Life Consortium are listed here: https://doi.org/10.5281/zenodo.4783558.
