# Supplementary material for: The genome sequence of the European Corn Borer, Ostrinia nubilalis Hübner, 1796
Source: Wellcome Open Res. 2025 Jan 14;10:12. [Version 1] doi: 10.12688/wellcomeopenres.23504.1 (PMC11851004; doi:10.12688/wellcomeopenres.23504.1)
Supplement: Peer review history

# Reviewer report: Reviewer response for version 1

**DOI:** 10.21956/wellcomeopenres.25924.r117296

**Published:** 2025-02-24

**Authors:** Himanshu Dubey

**Affiliations:** 1 CSB- Seri Biotech Research Laboratory, Bengaluru, Karnataka, India

The manuscript investigates the genome assembly of the European corn borer ( *Ostrinia nubilalis*), an economically significant pest species. It addresses a critical agricultural challenge by providing a comprehensive genomic resource to support future research on pest management and resistance mechanisms. The high-quality genome assembly, including chromosomal pseudomolecules and the mitochondrial genome, offers valuable insights for both functional and evolutionary studies. Overall, the manuscript is suitable for indexing.

Are sufficient details of methods and materials provided to allow replication by others?

Yes

Is the rationale for creating the dataset(s) clearly described?

Yes

Are the datasets clearly presented in a useable and accessible format?

Yes

Are the protocols appropriate and is the work technically sound?

Yes

Reviewer Expertise:

Genomics

I confirm that I have read this submission and believe that I have an appropriate level of expertise to confirm that it is of an acceptable scientific standard.

# Reviewer report: Reviewer response for version 1

**DOI:** 10.21956/wellcomeopenres.25924.r117297

**Published:** 2025-01-18

**Authors:** Arun Arumugaperumal, Achsha Babu

**Affiliations:** 1 Rajalakshmi Engineering College, Chennai, Tamilnadu, India; 2 Department of Biotechnology, Rajalakshmi Engineering College, Chennai, Tamil Nadu, India

**Background**

*Ostrinia nubilalis*, a moth belonging to Crambidae family originally native to Europe, Western Asia, and the Mediterranean, has emerged as a significant pest in North America, resulting in annual losses of over $1 billion in cultivated maize. While transgenic maize expressing Bt proteins suppressed populations since 1995, resistance to Cry1F and Cry1Ab proteins has emerged in recent years. This sexually dimorphic species shows ecological variation, including pheromone-mediated mate selection and latitudinal differences in reproductive cycles. Genetic factors like the autosomal gene pgfar and sex-linked genes bab, per, and pdfr, along with a Z-chromosome inversion, influence these adaptations. The study uses next-generation sequencing to assemble and annotate the genome, offering insights into the evolutionary and ecological dynamics of *O. nubilalis* and related species.

**Study Objectives**

 The study aims to elucidate the genetic underpinnings of *O. nubilalis* by sequencing its genome, which remains a significant challenge due to its complex biology. Understanding its genetics could lead to innovative pest control strategies, reducing dependence on chemical pesticides and supporting sustainable agriculture through integrated pest management (IPM).

**Methodology**

 Initially, samples were collected from Wytham Woods and High Wycombe, UK, with DNA barcoding using the cytochrome oxidase subunit 1 (COI) marker to confirm species identification. High quality DNA was extracted, homogenized, and purified using the MagAttract protocol, followed by fragmentation to 12–20 kb and quality assessment using Qubit and Nanodrop. Hi-C preparation involved tissue fixation, DNA crosslinking, and biotinylation, with subsequent library preparation using PacBio Express Kit for HiFi libraries and NEBNext Ultra II Kit for Hi-C libraries. The genome sequencing was conducted on PacBio Sequel IIe for HiFi libraries and Illumina NovaSeq 6000 for Hi-C libraries. The assembly was performed using Hifiasm and scaffolded with Hi-C data, with the mitochondrial genome reconstructed using MitoHiFi. Assembly curation was done using ASCC pipeline and PretextView, and genome quality was evaluated using BUSCO, Merqury.FK, and k-mer analysis. Annotation was conducted through Ensembl Genebuild using transcriptome alignment and UniProt protein-to-genome mappings.

**Key Findings**

 The genome of *O. nubilalis* was sequenced using Pacific Biosciences HiFi long reads, generating 21.96 Gb from 1.50 million reads, achieving 44-fold coverage. The primary assembly was scaffolded with 106.66 Gb of Hi-C data. After manual curation, including fixing 12 missing joins and 4 haplotypic duplications, the final assembly consists of 495.50 Mb in 52 scaffolds with a scaffold N50 of 16.5 Mb. The majority (99.87%) of the sequence was assigned to 32 chromosomal-level scaffolds, and the sex chromosomes were identified by coverage. The final assembly has a quality value of 68.8, with a k-mer completeness of 98.91%. BUSCO analysis indicated a completeness score of 98.5%. The genome was annotated with 45,650 transcribed mRNAs from 16,780 protein-coding and 10,413 non-coding genes, with an average transcript length of 13,875.99.

**Strength of the study**

 Using both short-read and long-read sequencing techniques increases the assembly quality, making the results more reliable and comprehensive.

**Limitation and future work**

 A deeper exploration of genome's complexity, especially its high proportion of repetitive sequences, may hinder transcriptomics or proteomics analysis. While the gene annotations offer theoretical insights, the lack of experimental validation limits conclusions about their roles in the biology of *O. nubilalis*. The comparative genomics approach deepens understanding of its evolutionary relationships with other Lepidoptera species and its ecological adaptability.

**Conclusion**

 This research provides a valuable contribution to genomics by delivering the complete genome of *O. nubilalis*, laying the foundation for studying its interactions with hosts and adaptation to environmental pressures, thereby advancing sustainable pest control strategies.

Are sufficient details of methods and materials provided to allow replication by others?

Yes

Is the rationale for creating the dataset(s) clearly described?

Yes

Are the datasets clearly presented in a useable and accessible format?

Yes

Are the protocols appropriate and is the work technically sound?

Yes

Reviewer Expertise:

Bioinformatics, Genomics

We confirm that we have read this submission and believe that we have an appropriate level of expertise to confirm that it is of an acceptable scientific standard.
